# Associations of Dietary Decanoic Acid Intake With Cognitive Function in the Elderly and the Mediating Effects of Hypertension and Diabetes: An Analysis From NHANES 2011–2014

**DOI:** 10.1002/brb3.71180

**Published:** 2026-01-28

**Authors:** Huangxin Zhu, Qingan Fu, Juanjuan Hu, Yihong Wu, Jun Min

**Affiliations:** ^1^ Department of Neurology The Second Affiliated Hospital of Nanchang University Nanchang Jiangxi China; ^2^ Department of Gastroenterology The Second Affiliated Hospital of Nanchang University Nanchang Jiangxi China; ^3^ Department of Cardiovascular Medicine The Second Affiliated Hospital of Nanchang University Nanchang Jiangxi China

**Keywords:** cognitive function, dietary decanoic acid, mediating effect, medium‐chain fatty acids, NHANES

## Abstract

**Background:**

Cognitive decline among the elderly is an increasingly prominent issue amid global population aging. Decanoic acid has been hypothesized to be associated with cognitive function. However, the relationship of decanoic acid with cognitive function in the elderly remains unclear.

**Methods:**

This study analyzed the participants aged 60 years and older from National Health and Nutrition Examination Survey (NHANES) 2011–2014. Dietary decanoic acid (DDA) intake was derived from two 24‐h dietary recalls. Cognitive function was assessed via immediate recall test (IRT), delayed recall test (DRT), animal fluency test (AFT), and digit symbol substitution test (DSST). Higher scores on these tests indicated better cognitive performance. Weighted multivariate linear regression, restricted cubic spline (RCS) curves, subgroup analyses, and mediating analysis were used to explore the relationship between DDA intake and cognitive function.

**Results:**

A total of 2246 older adults were included in this study. After adjusting for confounding variables, DDA intake was positively associated with comprehensive cognitive function (*β* = 0.539, 95% CI: 0.168–0.910, *p* = 0.007). The RCS curve shows a positive correlation between DDA intake and comprehensive cognitive function (*p*‐value for overall < 0.001, *p*‐value for nonlinearity = 0.050). Subgroup analyses showed that the association remained relatively consistent across subgroups (all *p* for interaction > 0.05). Mediating analysis revealed that the indirect effects of hypertension and diabetes accounted for 27.53% and 24.33% of the total effect, respectively.

**Conclusion:**

DDA intake is positively associated with global cognitive function in older adults. Hypertension and diabetes may partially mediate this relationship. The cross‐sectional study design limits causal inference, and prospective or interventional studies should be conducted in the future.

## Graphical abstract

This study included elderly people aged 60 and above in the NHANES 2011–2014 cycle. The research indicated that increased dietary decanoic acid intake was associated with better cognitive function in the elderly, among which diabetes and hypertension were statistically associated with a partial mediating effect, although causality cannot be inferred.

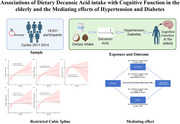

## 1 Introduction

As global population aging accelerates, individuals aged 60 years and older are projected to account for 22% of the world's population by 2050 (Beard et al. 2016). Against this backdrop, cognitive deterioration is becoming an increasingly prominent public‐health concern (Wang et al. 2022). Cognitive decline constitutes a transitional stage between normal cognition and dementia, during which individuals largely retain everyday functioning (Hugo and Ganguli 2014). This decline may stem from degenerative changes in brain structure and function, including neuronal damage, neurotransmitter alterations, and cerebrovascular disease (Flesch et al. 2015). Cognitive decline is associated with multiple factors, including chronic disease, lifestyle, genetics, and the social environment (Tsuang et al. 2013; Brown et al. 2005). Cognitive decline in older adults typically manifests as memory loss, poor concentration, and executive dysfunction (Ruan et al. 2015). These symptoms can impair activities of daily living and increase the risk of adverse health outcomes such as falls, hospitalization, and disability (Zhang et al. 2022). Without effective intervention, cognitive decline may eventually progress to dementia, an irreversible condition (Campbell et al. 2013). Therefore, early identification and intervention are crucial for delaying cognitive decline. To meet this challenge, interventions including pharmacotherapy, cognitive training, and lifestyle modification have been implemented (Mangialasche et al. 2010; Cui et al. 2018; Dominguez et al. 2021). Research has shown that specific dietary components significantly preserve cognitive abilities and mitigate the risk of cognitive decline (Chu et al. 2022; Poulose et al. 2017).

Medium‐chain fatty acids (MCFAs)—saturated fatty acids with 6–12 carbon atoms—are crucial for energy metabolism and human health (Huang et al. 2021). Although some studies have examined the impact of MCFAs on cognitive performance, stronger associations with cognitive health have been reported for short‐chain fatty acids (SCFAs) and long‐chain omega‐3 fatty acids. SCFAs regulate gut–microbiota metabolism and thereby influence cognition via the gut–brain axis (Qian et al. 2022; Guo et al. 2023). Increased intake of omega‐3 polyunsaturated fatty acids may reduce the risk that older adults with mild cognitive impairment will progress to dementia (Sinn et al. 2012). Decanoic acid (C10:0) is an MCA with ten carbon atoms; its natural sources are limited and include milk fat, coconut oil, and palm‐kernel oil (Jadhav and Annapure 2023; Jensen 2002). Multiple studies suggest that decanoic acid may reduce the incidence of coronary artery disease and epilepsy, and delay progression from prediabetes to diabetes (Wu et al. 2023; Chang et al. 2016; Han et al. 2021; Zhu et al. 2024). Furthermore, decanoic acid can alleviate neuropathic pain and improve affective disorders and Parkinson's disease to some extent (Nakajima et al. 2018; Shoji et al. 2022; Sanguanphun et al. 2022). Based on this evidence, we believe that decanoic acid may have a neuroprotective effect. We therefore hypothesize that higher dietary decanoic acid (DDA) intake is associated with better cognitive function and may offer a novel dietary strategy for cognitive health. Currently, no large‐scale population‐based study has investigated the link between DDA intake and cognitive function. In addition, decanoic acid is thought to improve endothelial function, alleviate insulin resistance, and ameliorate hypertension and diabetes—conditions known to contribute to cognitive deterioration in older adults (Chakraborty et al. 2018; Nonaka et al. 2022). Diabetes and hypertension may therefore mediate the association between DDA intake and cognitive function.

In this study, we obtained data on DDA intake and cognitive tests from the 2011–2014 cycles of the National Health and Nutrition Examination Survey (NHANES). The objective was to examine the association between DDA intake and cognitive function among US older adults and to assess whether hypertension and diabetes mediate this relationship.

## 2 Methods

### 2.1 Study Design and Population

The NHANES is a nationally representative health and nutrition survey conducted by the Centers for Disease Control and Prevention (CDC) in the United States. This cross‐sectional survey is designed to assess the health and nutritional status of the US population, including both adults and children. Extensive health and nutrition data are collected using a multistage probability sampling design across continuous survey cycles. Participants are invited to the Mobile Examination Center (MEC) for physical examinations and collection of biological specimens. In addition, participants undergo household interviews to provide information on dietary habits, physical activity, and healthcare utilization. The National Center for Health Statistics Research Ethics Review Board approved the survey. Informed consent was obtained from all individuals participating in the study. Therefore, no additional Institutional Review Board approval was required for the present analysis. The NHANES data used in this study are publicly available and can be accessed free of charge at https://wwwn.cdc.gov/nchs/nhanes/Default.aspx.

**FIGURE 1:**
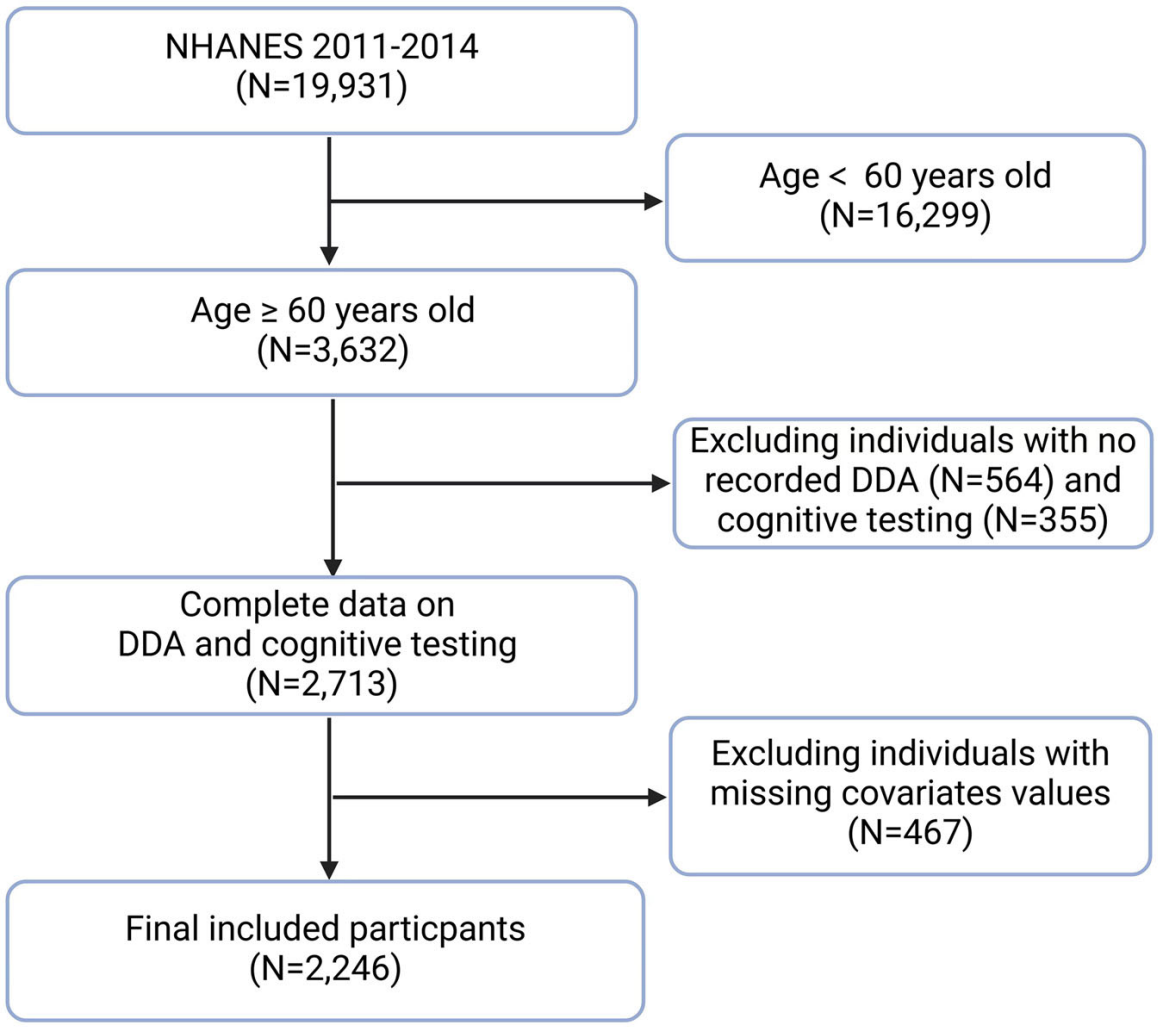
The flow chart of inclusion and exclusion criteria in the study. Abbreviations: NHANES, National Health and Nutrition Examination Survey; DDA, dietary decanoic acid.

We used NHANES data from the 2011–2014 cycles (two survey cycles) for this analysis. These cycles included 19,931 participants. Following the exclusion criteria shown in Figure 1, we excluded participants who were aged < 60 years (*n* = 16,299), lacked DDA data (*n* = 564), lacked cognitive test data (*n* = 355), or lacked key covariate data (*n* = 467). Thus, the final analytic sample comprised 2246 participants.

### 2.2 Measurement of Dietary Decanoic Acid

The NHANES dietary component records detailed intake data for all participants. Dietary intake was assessed using two 24‐h recalls per participant. The first recall was conducted in person at the MEC, and the second by telephone 3–10 days later. For the present analysis, DDA intake was estimated by averaging the two recalls.

### 2.3 Evaluation of Cognitive Performance

Cognitive function in adults aged 60 and above was assessed with four tests: immediate recall test (IRT), delayed recall test (DRT), animal fluency test (AFT), and digit symbol substitution test (DSST) (Fu et al. 2025). These tests were administered face‐to‐face at the MEC. Before testing began, interviewers obtained written informed consent from participants; sessions were audio‐recorded. The AFT evaluates language and word‐retrieval ability and serves as an early screen for dementia and mild cognitive impairment (Clark et al. 2009). During the test, participants have 60 s to name as many animals as possible. One point is awarded for each valid name. The DSST assesses multiple cognitive domains, including attention, processing speed, working memory, and executive function (Holtzer et al. 2012). In the paper‐and‐pencil version, nine digits are each paired with a unique symbol. Participants match the corresponding symbol in 133 empty boxes within 2 min. The score equals the number of correct matches. IRT and DRT are components of the Consortium to Establish a Registry for Alzheimer's Disease (CERAD) word‐learning task, designed to assess immediate and delayed memory (Fillenbaum et al. 2008). The IRT comprises three learning trials in which participants immediately recall a list of 10 unrelated words after each presentation. The IRT score is the sum of the words recalled across the three trials. For the DRT, participants recall the same 10 words approximately 8–10 min after the first IRT trial (after completing IRT, AFT, and DSST). Each correctly recalled word counts as one point.

Higher scores indicate better cognitive performance. To facilitate comparison across tests, raw scores were converted to Z‐score, providing a standardized metric. The z‐score was computed as *Z* = (*x* — *μ*)/*σ*, where *x* is the individual raw score, μ the test‐specific mean, and σ the standard deviation. A comprehensive cognitive Z‐score was obtained by averaging the four individual Z‐scores, and served as an index of overall cognitive performance.

### 2.4 Potential Covariates

Covariates considered included some of the following demographic characteristics, dietary data, body measurements, laboratory tests and questionnaires: gender (male/female), age (< 70 years/≥ 70 years), race (Mexican American/non‐Hispanic White/Non‐Hispanic Black/other race), education level (high school or below/college or above), economy defined by the income‐to‐poverty ratio (PIR < 1.0/PIR ≥ 1.0), marital status (married/living with partner, widowed/divorced/separated, never married), dietary energy (kcal/d), protein (g/d) and carbohydrates (g/d), body mass index (BMI, kg/m^2^), waist circumference (WC, cm), triglyceride (TG, mmol/L), total cholesterol (TC, mmol/L). Smoking status was determined based on the response to the query, “Have you smoked at least a hundred cigarettes in your lifetime?” Participants were classified as either having smoked (Yes) or not smoked (No). The drinking status of participants is determined by whether their annual alcohol consumption exceeds 12 drinks, which is considered as Yes or No. Hypertension can be diagnosed if any of the following conditions are met, including a systolic blood pressure (SBP) of ≥ 130 mmHg and/or a diastolic blood pressure (DBP) of ≥ 80 mmHg, or if the doctor has informed that you have hypertension, or if you are currently taking antihypertensive medication (Whelton et al. 2018). A person is considered to be suffering from stroke if he or she answers “Yes” to the question: “Have you been told you had a stroke?” Diabetes was identified as having a fasting blood glucose (FBG) level of 126 mg/dL or higher, or a glycated hemoglobin A1c (HbA1c) level of 6.5% or higher, or by affirming any of the following inquiries: “Have you been diagnosed with diabetes by a healthcare professional?,” “Are you currently on insulin therapy?,” and “Are you currently using oral hypoglycemic medications?”

### 2.5 Statistical Analysis

In this study, we utilized the R software environment to carry out the statistical analyses (https://www.r‐project.org/; version 4.3.3). This study followed the NHANES analysis guidelines. The “WTMEC2YR” weights were selected for the weighted analysis to address the complex sampling design, and the “survey” R package was used for the analysis (Xiao et al. 2025). We divided the DDA intake of participants into quartiles, using the first quartile as the comparative reference (Q1). The baseline characteristics of study participants were delineated based on their DDA intake quartiles. The continuous variables are examined using Mann–Whitney *U* tests and expressed as median and quartiles. The categorical variables are compared using *Chi‐square* test, and described by number (*n*) and percentage (%). A *p*‐value of less than 0.05 from a two‐tailed test was considered to indicate statistical significance.

Weighted multivariate linear regression analysis was employed to examine the association between DDA intake and cognitive function across three distinct models, each with a different set of covariates for adjustment. Model 1 adjusts for no variables. Model 2 incorporated adjustments for gender, age, race, education levels, PIR, and marital status. Building on Model 2, Model 3 additionally accounted for BMI, WC, TC, TG, drinking status, smoking status, stroke, hypertension, and diabetes. The dose–response relationship between DDA intake and cognitive function was depicted using a restricted cubic spline (RCS) curve in the fully adjusted Model 3. The analysis results were presented in terms of the beta coefficient (*β*) along with its respective 95% confidence interval (95% CI).

Subgroup analysis was used to evaluate the consistency of the association between DDA intake and comprehensive cognitive function in the elderly across different populations. Subgroups include demographics, BMI, intake of different nutrients and comorbidities. Demographics includes gender (male/female), age (< 70 years/≥ 70 years), and educational level (high school or below/college or above). BMI is classified into obese (BMI ≥ 30 kg/m^2^) and non‐obese (BMI < 30 kg/m^2^). Participants were divided into a low‐intake group and a high‐intake group based on the median intake of protein, energy, and carbohydrates. Complications included hypertension (Yes/No), diabetes (Yes/No), and stroke (Yes/No).

To explore whether diabetes/hypertension mediates the association between DDA and comprehensive cognitive function, we conducted a mediating effect analysis. Hypertension and diabetes were tested in two separate mediating analyses: DDA‐hypertension‐comprehensive cognition and DDA‐diabetes‐comprehensive cognition. This strategy can avoid collinearity between the two comorbidities and make the explanation of the indirect effects specific to each mediator more transparent. The “mediation” R package was used for mediating effect analysis with the causal‐steps approach outlined by Baron and Kenny together, and 1000 nonparametric bootstrap iterations were adopted to estimate the indirect effect (mediating effect) (Yin et al. 2025). Firstly, two regression models were constructed: the mediating model and the outcome model (Zhu et al. 2025). In the mediating model, diabetes/hypertension is taken as the dependent variable, DDA as the exposure variable, and all covariates in Model 3 are simultaneously included. Weighted multivariate logistic regression was used to estimate the impact of DDA on diabetes/hypertension. In the outcome model, the comprehensive Z‐score is taken as the dependent variable, while DDA and diabetes/hypertension are used as predictors, and the same covariates in the mediating model are retained. In the analysis of hypertension, diabetes was controlled, and in the analysis of diabetes, hypertension was controlled. The direct impact of DDA on the comprehensive Z‐score and the impact of diabetes/hypertension on the comprehensive Z‐score were evaluated, respectively, through multivariate linear regression.

To verify the robustness of the association between DDA intake and cognitive function in the elderly, we conducted a sensitivity analysis: the DDA intake was calculated using only the 24‐h dietary retrospective questionnaire on the first day instead of the two‐day average. Weighted multivariate linear regression with the same covariate structure was adopted, and the results were compared with the two‐day average DDA intake.

## 3 Results

### 3.1 Baseline Characteristics

**FIGURE 2:**
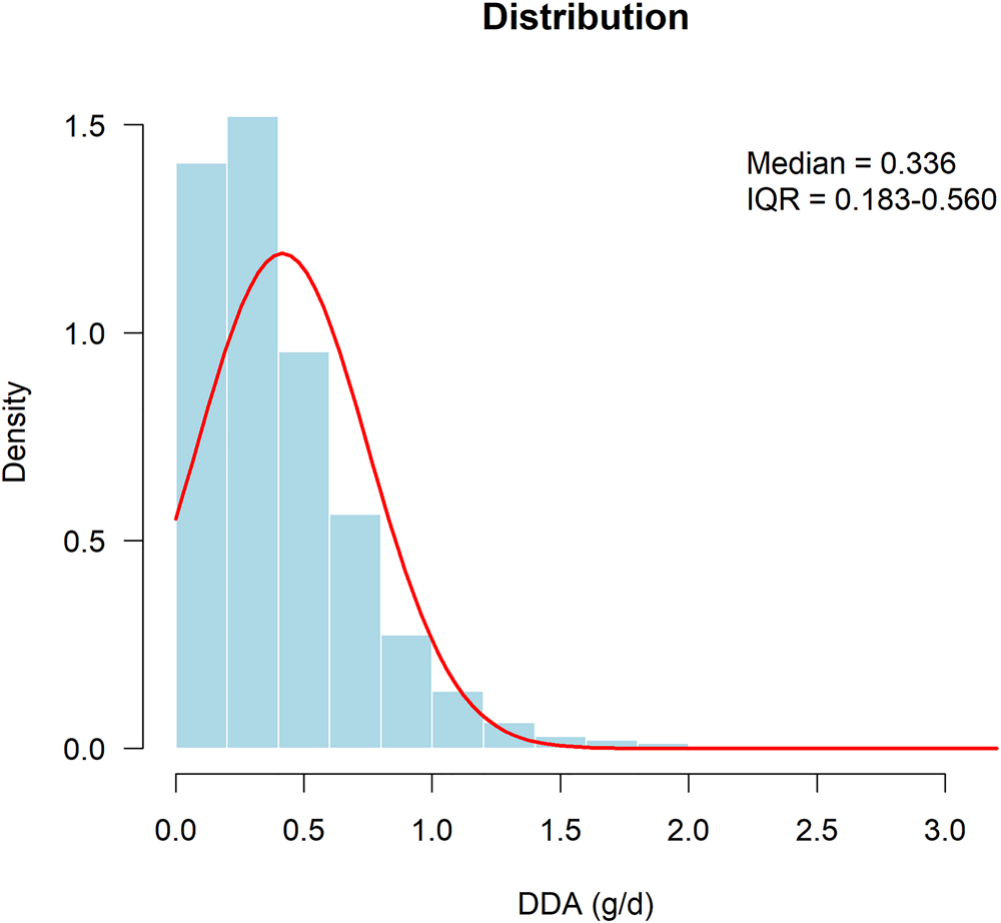
The distribution of DDA intake.

A total of 2246 eligible participants aged 60 and above were included in this study, with a median DDA intake of 0.336 (0.183–0.560) g/d (Figure 2). The weighted results show that these people represent 42,015,992 elderly people of the same age group in the United States, which is basically consistent with previous research reports (Wu et al. 2023). Table 1 shows the baseline characteristics of participants according to the quartiles of DDA intake. Among these participants, 50.6% were female, over half (55.6%) were under 70 years old, 50.7% were non‐Hispanic White, 53.3% had a college education or above, 15.6% were economically poor, and 59.2% were married or cohabiting. Compared with the lowest group (Q1), the proportion of males in the highest intake group (Q4) increased by more than 10 percentage points (43.9% vs. 55.5%), the proportion of non‐Hispanic Whites jumped from 33.6% to 65.3%, and the proportion of those with a college education or above increased by 20 percentage points (41.9% vs. 61.6%). The number of people with income above the poverty line increased by nearly 10% (79.2% vs. 86.1%), the prevalence of diabetes decreased by nearly 10% (36.6% vs. 25.6%), and the prevalence of hypertension dropped by 7% (82.9% vs. 75.6%). The most notable gap was reflected in the cognitive score: The median of the comprehensive cognitive Z‐score rose from –0.38 to +0.75, spanning 1.13 standard deviations.

**TABLE 1:** Baseline characteristics of participants according to the quartiles of DDA intake.

		Quartiles of DDA intake, g/d				*p*‐value
Variables	Total *n* = 2246	Q1 (< 0.183) *n* = 563	Q2 (0.183–0.336) *n* = 562	Q3 (0.336–0.560) *n* = 559	Q4 (≥ 0.560) *n* = 562	
**Gender, *n* (%)**						<0.001
Female	1136 (50.6)	316 (56.1)	310 (55.2)	260 (46.5)	250 (44.5)	
Male	1110 (49.4)	247 (43.9)	252 (44.8)	299 (53.5)	312 (55.5)	
**Age, *n* (%)**						0.275
< 70 years	1249 (55.6)	321 (57.0)	327 (58.2)	297 (53.1)	304 (54.1)	
≥ 70 years	997 (44.4)	242 (43.0)	235 (41.8)	262 (46.9)	258 (45.9)	
**Race, *n* (%)**						<0.001
Mexican American	198 (8.8)	55 (9.8)	51 (9.1)	55 (9.8)	37 (6.6)	
Non‐Hispanic White	1138 (50.7)	189 (33.6)	262 (46.6)	320 (57.2)	367 (65.3)	
Non‐Hispanic Black	499 (22.2)	179 (31.8)	125 (22.2)	107 (19.1)	88 (15.7)	
Other races	411 (18.3)	140 (24.9)	124 (22.1)	77 (13.8)	70 (12.5)	
**Education level, *n* (%)**						<0.001
High school or below	1048 (46.7)	327 (58.1)	276 (49.1)	229 (41.0)	216 (38.4)	
College or above	1198 (53.3)	236 (41.9)	286 (50.9)	330 (59.0)	346 (61.6)	
**Economy, *n* (%)**						<0.001
PIR < 1	350 (15.6)	117 (20.8)	90 (16.0)	65 (11.6)	78 (13.9)	
PIR ≥ 1	1896 (84.4)	446 (79.2)	472 (84.0)	494 (88.4)	484 (86.1)	
**Marital status, *n* (%)**						0.672
Married/Living with a partner	1329 (59.2)	319 (56.7)	343 (61.0)	323 (57.8)	344 (61.2)	
Widowed/divorced/separated	791 (35.2)	208 (36.9)	190 (33.8)	205 (36.7)	188 (33.5)	
Never married	126 (5.6)	36 (6.4)	29 (5.2)	31 (5.5)	30 (5.3)	
**BMI, kg/m2**	28.10 (24.80, 32.38)	28.00 (24.60, 33.00)	28.00 (24.90, 32.20)	28.60 (25.20, 32.75)	28.00 (24.70, 31.50)	0.252
**BMI, *n* (%)**						0.168
< 30 (kg/m2)	1389 (61.8)	345 (61.3)	357 (63.5)	326 (58.3)	361 (64.2)	
≥ 30 (kg/m2)	857 (38.2)	218 (38.7)	205 (36.5)	233 (41.7)	201 (35.8)	
**WC, cm**	101.30 (92.53, 110.88)	100.40 (91.50, 110.65)	100.45 (92.55, 109.57)	102.30 (93.40, 112.70)	102.15 (92.70, 110.65)	0.103
**Smoking status, *n* (%)**						0.048
No	1095 (48.8)	302 (53.6)	260 (46.3)	260 (46.5)	273 (48.6)	
Yes	1151 (51.2)	261 (46.4)	302 (53.7)	299 (53.5)	289 (51.4)	
**Drinking status, *n* (%)**						<0.001
No	669 (29.8)	210 (37.3)	165 (29.4)	154 (27.5)	140 (24.9)	
Yes	1577 (70.2)	353 (62.7)	397 (70.6)	405 (72.5)	422 (75.1)	
**Diabetes, *n* (%)**						<0.001
No	1568 (69.8)	357 (63.4)	385 (68.5)	408 (73.0)	418 (74.4)	
Yes	678 (30.2)	206 (36.6)	177 (31.5)	151 (27.0)	144 (25.6)	
**Hypertension, *n* (%)**						0.009
No	452 (20.1)	96 (17.1)	101 (18.0)	118 (21.1)	137 (24.4)	
Yes	1794 (79.9)	467 (82.9)	461 (82.0)	441 (78.9)	425 (75.6)	
**Stroke, *n* (%)**						0.931
No	2097 (93.4)	529 (94.0)	523 (93.1)	521 (93.2)	524 (93.2)	
Yes	149 (6.6)	34 (6.0)	39 (6.9)	38 (6.8)	38 (6.8)	
**IRT Z‐score**	0.18 (–0.71, 0.62)	−0.04 (–0.71, 0.62)	−0.04 (–0.71, 0.62)	0.18 (–0.60, 0.84)	0.18 (–0.49, 0.84)	<0.001
**DRT Z‐score**	−0.03 (–0.47, 0.85)	−0.03 (–0.47, 0.85)	−0.03 (–0.47, 0.85)	−0.03 (–0.47, 0.85)	−0.03 (–0.47, 0.85)	0.170
**AFT Z‐score**	−0.18 (–0.73, 0.55)	−0.36 (–0.91, 0.37)	−0.18 (–0.73, 0.55)	0.00 (–0.73, 0.73)	0.19 (–0.36, 0.73)	<0.001
**DSST Z‐score**	−0.02 (–0.73, 0.70)	−0.25 (–0.97, 0.40)	0.04 (–0.79, 0.76)	0.10 (–0.67, 0.81)	0.16 (–0.43, 0.87)	<0.001
**Comprehensive Z‐score**	0.11 (–2.21, 2.25)	−0.38 (–2.84, 1.41)	−0.15 (–2.39, 2.23)	0.36 (–1.96, 2.44)	0.75 (–1.40, 2.80)	<0.001
**TC, mmol/L**	4.89 (4.11, 5.64)	4.89 (4.11, 5.64)	4.96 (4.16, 5.79)	4.86 (4.07, 5.60)	4.81 (4.09, 5.59)	0.260
**TG, mmol/L**	1.43 (0.96, 2.16)	1.40 (0.95, 2.03)	1.45 (0.97, 2.28)	1.46 (0.97, 2.16)	1.42 (0.96, 2.16)	0.655
**Energy, kcal/d**	1753.50 (1351.75, 2218.12)	1366.00 (1028.00, 1729.50)	1620.25 (1254.00, 1978.38)	1849.00 (1533.50, 2293.50)	2210.75 (1813.75, 2694.88)	<0.001
**Protein, g/d**	69.58 (52.97, 88.49)	56.61 (41.16, 75.84)	63.90 (51.22, 81.31)	72.27 (57.49, 90.12)	83.49 (67.02, 104.04)	<0.001
**Carbohydrate, g/d**	213.31 (161.00, 274.43)	172.06 (129.08, 231.93)	194.86 (150.07, 249.03)	222.48 (175.99, 280.97)	262.67 (206.42, 326.42)	<0.001

*Note*: The continuous variables were expressed as median and quartiles, and the categorical variables were expressed as number and percentage.

Abbreviations: DDA, dietary decanoic acid; PIR, poverty income ratio; BMI, body mass index; WC, waist circumference; TG, triglyceride; TC, total cholesterol.

### 3.2 Association Between DDA and Cognitive Performance in Elderly

Table 2 presents a summary of the findings from the weighted multivariate linear regression analysis, which investigated the relationship between continuous and quartile‐transformed DDA and cognitive performance among the elderly population. The continuous analysis showed, in Model 1 without adjusting for other variables, DDA intake was significantly positively correlated with Z‐scores of four tests and comprehensive cognition. In the fully adjusted Model 3, except for the AFT Z‐score, the association between DDA intake and the Z‐scores of other cognitive tests and the comprehensive Z‐score still maintained a significantly positive relationship. Specifically, for every 1 g/d increase in DDA intake, it is associated with an increase of 0.227 units in the average IRT Z‐score (*β* = 0.227, 95% CI: 0.086–0.367, *p* = 0.004), 0.141 units in the average DRT Z‐score (*β* = 0.141, 95% CI: 0.018–0.264, *p* = 0.027), 0.130 units in the average DSST Z‐score (*β* = 0.130, 95% CI: 0.028–0.233, *p* = 0.016), and 0.539 units in the average comprehensive Z‐score (*β* = 0.539, 95% CI: 0.168–0.910, *p* = 0.007). The quartile analysis revealed that the highest quartile of DDA intake was linked to superior cognitive performance in comparison to the lowest quartile. As the quartiles of DDA increased, cognitive performance also significantly increased accordingly.

**TABLE 2:** Weighted multivariate linear regression analysis between DDA intake and cognitive function.

DDA, g/d	Model 1		Model 2		Model 3	
	*β* (95% CI)	*p*‐value	*β* (95% CI)	*p*‐value	*β* (95% CI)	*p*‐value
**IRT Z‐scores**						
Continuous	0.274 (0.151, 0.397)	<0.001	0.249 (0.121, 0.376)	<0.001	0.227 (0.086, 0.367)	0.004
Quartiles						
Q1	Ref		Ref		Ref	
Q2	0.231 (0.106, 0.355)	<0.001	0.185 (0.047, 0.323)	0.011	0.174 (0.021, 0.327)	0.029
Q3	0.213 (0.037, 0.389)	0.019	0.167 (–0.002, 0.336)	0.053	0.151 (–0.031, 0.334)	0.097
Q4	0.343 (0.213, 0.474)	<0.001	0.311 (0.166, 0.455)	<0.001	0.288 (0.127, 0.450)	0.002
*p* for trend		<0.001		<0.001		0.003
**DRT Z‐scores**						
Continuous	0.175 (0.057, 0.294)	0.005	0.161 (0.046, 0.276)	0.008	0.141 (0.018, 0.264)	0.027
Q1	Ref		Ref		Ref	
Q2	0.151 (0.017, 0.286)	0.029	0.118 (–0.009, 0.245)	0.066	0.105 (–0.024, 0.233)	0.103
Q3	0.153 (–0.033, 0.340)	0.103	0.125 (–0.052, 0.302)	0.157	0.106 (–0.079, 0.292)	0.239
Q4	0.232 (0.106, 0.359)	<0.001	0.216 (0.105, 0.327)	<0.001	0.197 (0.074, 0.319)	0.004
*p* for trend		0.003		0.003		0.012
**AFT Z‐scores**						
Continuous	0.251 (0.082, 0.420)	0.005	0.068 (–0.101, 0.236)	0.417	0.041 (–0.129, 0.211)	0.614
Q1	Ref		Ref		Ref	
Q2	0.243 (0.077, 0.408)	0.006	0.145 (0.012, 0.278)	0.034	0.130 (–0.011, 0.271)	0.069
Q3	0.307 (0.107, 0.507)	0.004	0.144 (–0.021, 0.310)	0.085	0.125 (–0.048, 0.299)	0.144
Q4	0.350 (0.150, 0.550)	0.001	0.164 (–0.013, 0.342)	0.068	0.134 (–0.048, 0.316)	0.137
*p* for trend		0.001		0.107		0.187
**DSST Z‐scores**						
Continuous	0.288 (0.167, 0.409)	<0.001	0.163 (0.069, 0.258)	0.001	0.130 (0.028, 0.233)	0.016
Q1	Ref		Ref		Ref	
Q2	0.265 (0.119, 0.410)	<0.001	0.167 (0.067, 0.268)	0.002	0.146 (0.038, 0.255)	0.012
Q3	0.260 (0.099, 0.422)	0.003	0.116 (–0.022, 0.254)	0.096	0.089 (–0.042, 0.219)	0.168
Q4	0.371 (0.213, 0.529)	<0.001	0.232 (0.125, 0.340)	<0.001	0.192 (0.082, 0.301)	0.002
*p* for trend		<0.001		0.002		0.009
**Comprehensive Z‐scores**						
Continuous	0.988 (0.640, 1.336)	<0.001	0.641 (0.302, 0.980)	<0.001	0.539 (0.168, 0.910)	0.007
Q1	Ref		Ref		Ref	
Q2	0.890 (0.454, 1.325)	<0.001	0.615 (0.284, 0.947)	<0.001	0.555 (0.184, 0.927)	0.006
Q3	0.934 (0.365, 1.503)	0.002	0.552 (0.054, 1.050)	0.031	0.471 (–0.054, 0.997)	0.075
Q4	1.297 (0.859, 1.735)	<0.001	0.924 (0.566, 1.281)	<0.001	0.810 (0.416, 1.205)	<0.001
*p* for trend		<0.001		<0.001		0.001

*Note*: Model 1 is unadjusted. Model 2 adjusts for gender, age, race, education level, economy, and marital status. Model 3 adjusts for gender, age, race, education level, economy, marital status, BMI, WC, TC, TG, drinking status, smoking status, stroke, hypertension, and diabetes.

### 3.3 Dose–Response Relationship Between DDA and Cognitive Function in Elderly

**FIGURE 3:**
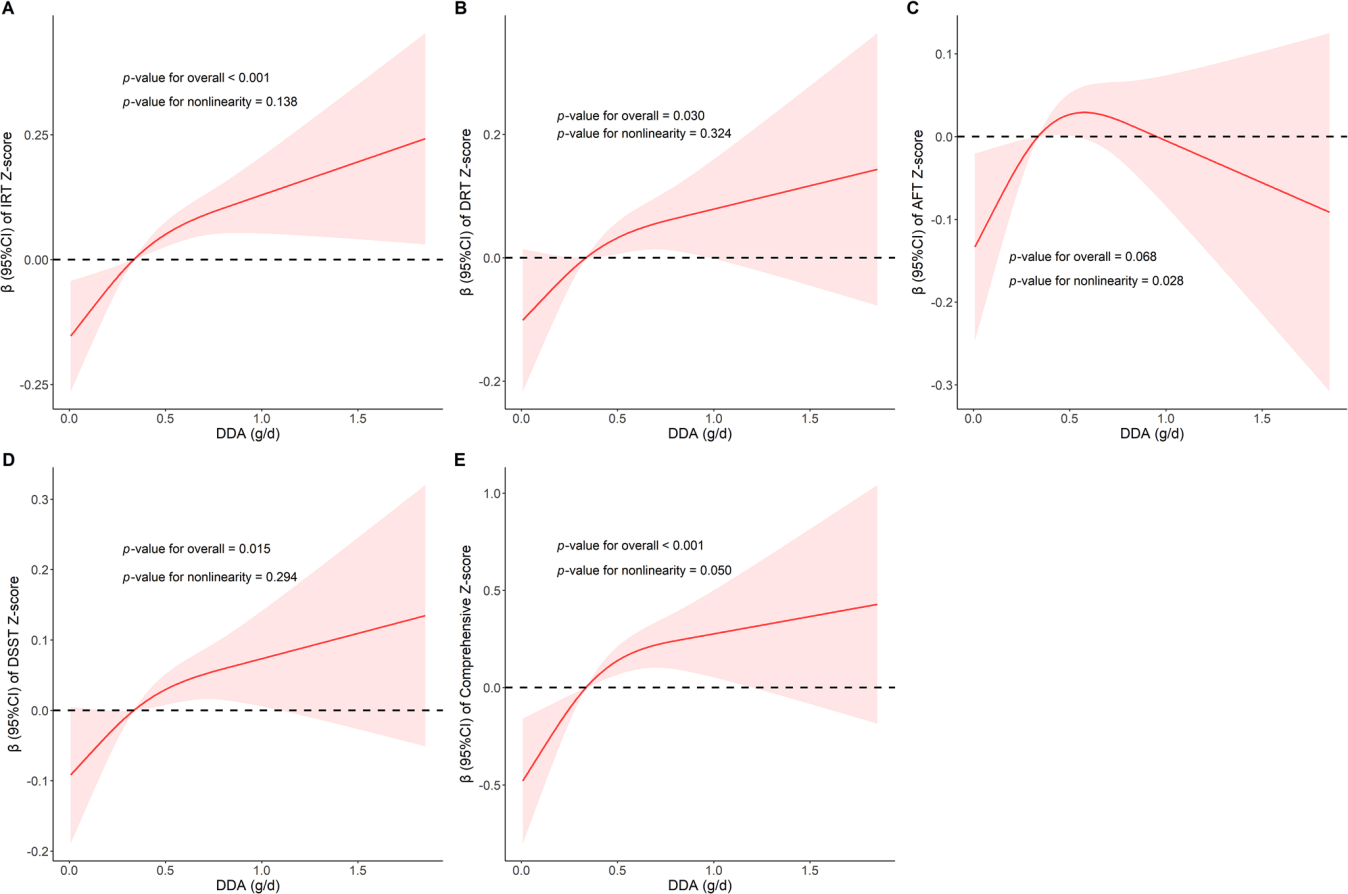
The dose–response relationship of DDA intake with the IRT Z‐score (A), DRT Z‐score (B), AFT Z‐score (C), DSST Z‐score (D), and comprehensive Z‐score (E). The *β* (red solid lines) and 95% CI (red shaded areas) in the RCS was adjusted for gender, age, race, education level, economy, marital status, BMI, WC, TC, TG, drinking status, smoking status, stroke, hypertension, and diabetes.

In fully adjusted Model 3, the RCS analysis was conducted to explore the dose–response relationship between DDA intake and cognitive function (Figure 3). The results indicate that in the RCS curves of AFT Z‐score (*p*‐value for nonlinearity = 0.028) and comprehensive cognition (*p*‐value for nonlinearity = 0.050), they show a trend of first increasing and then gradually flattening or decreasing with the increase of DDA intake. The gentle or downward part at the end of the RCS curve involves very few participants (Figure 2) and an extremely wide 95% CI band (Figure 3), which may reflect the sparsity of the data rather than a true biological plateau. Our results also suggested that the relationship between DDA intake and the IRT Z‐score (*p*‐value for nonlinearity = 0.138), DRT Z‐scores (*p*‐value for nonlinearity = 0.324) and DSST Z‐score (*p*‐value for nonlinearity = 0.294) may be closer to linear and more direct, and this effect is relatively stable within the range of DDA intake examined.

### 3.4 Subgroup Analysis

**FIGURE 4:**
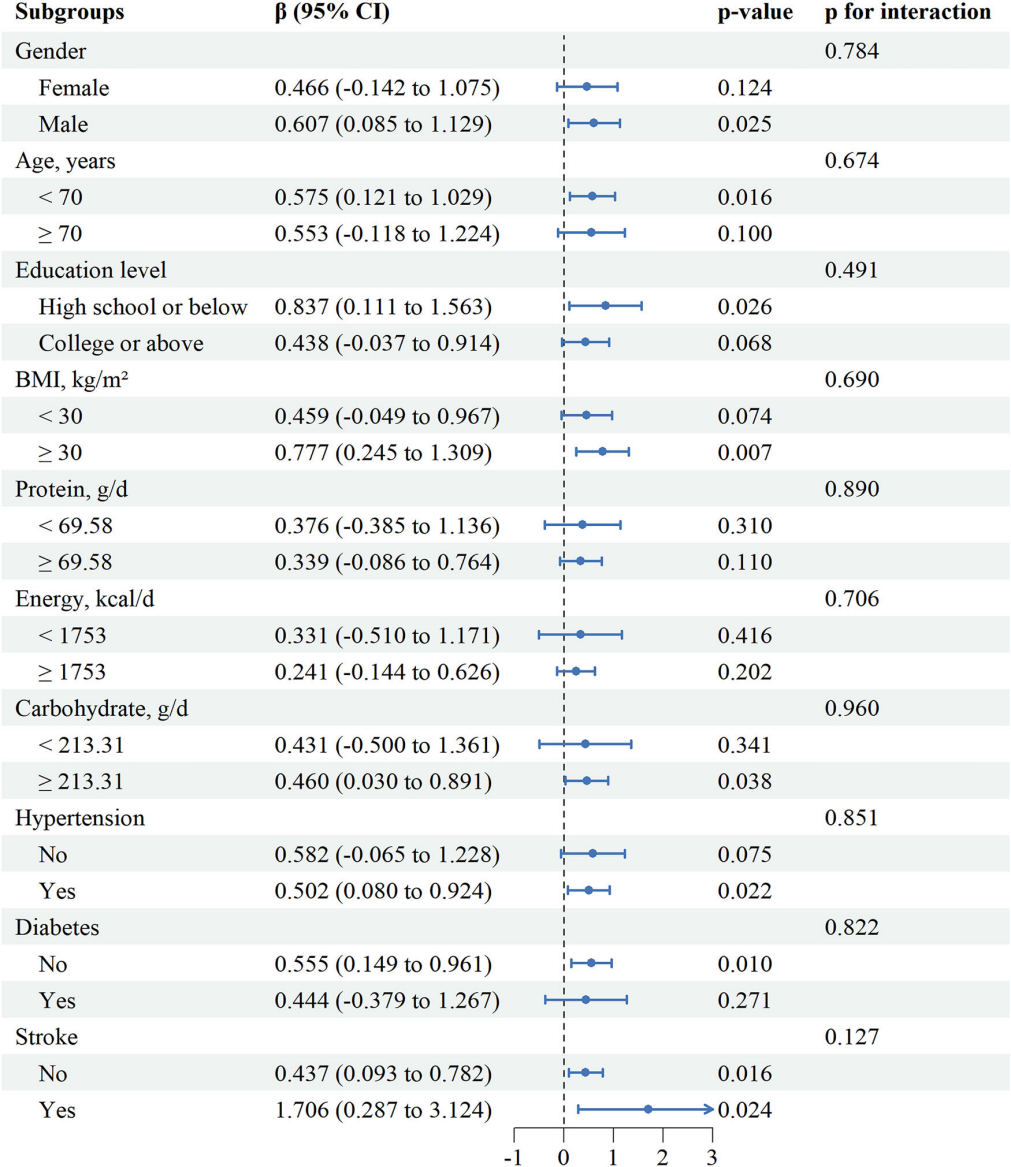
Subgroup analysis of association between DDA intake and comprehensive cognitive function. Each subgroup analysis was adjusted for gender, age, race, education level, economy, marital status, BMI, WC, TC, TG, drinking status, smoking status, stroke, hypertension, and diabetes. The stratified variable was not included when stratifying by itself.

We conducted subgroup analyses of demographics, BMI, nutrient intake, and comorbidities in Figure 4. The results show that the positive correlation between DDA intake and comprehensive cognitive function remains significant among men under 70 years old, those with high school education or below, obese individuals, those with high carbohydrate intake, hypertension, non‐diabetes, stroke and non‐stroke populations. However, no significant interaction was observed in any subgroup. This indicates that in the current research, the impact of DDA intake on comprehensive cognitive function is basically the same in different subgroups.

### 3.5 Mediating Analysis

**FIGURE 5:**
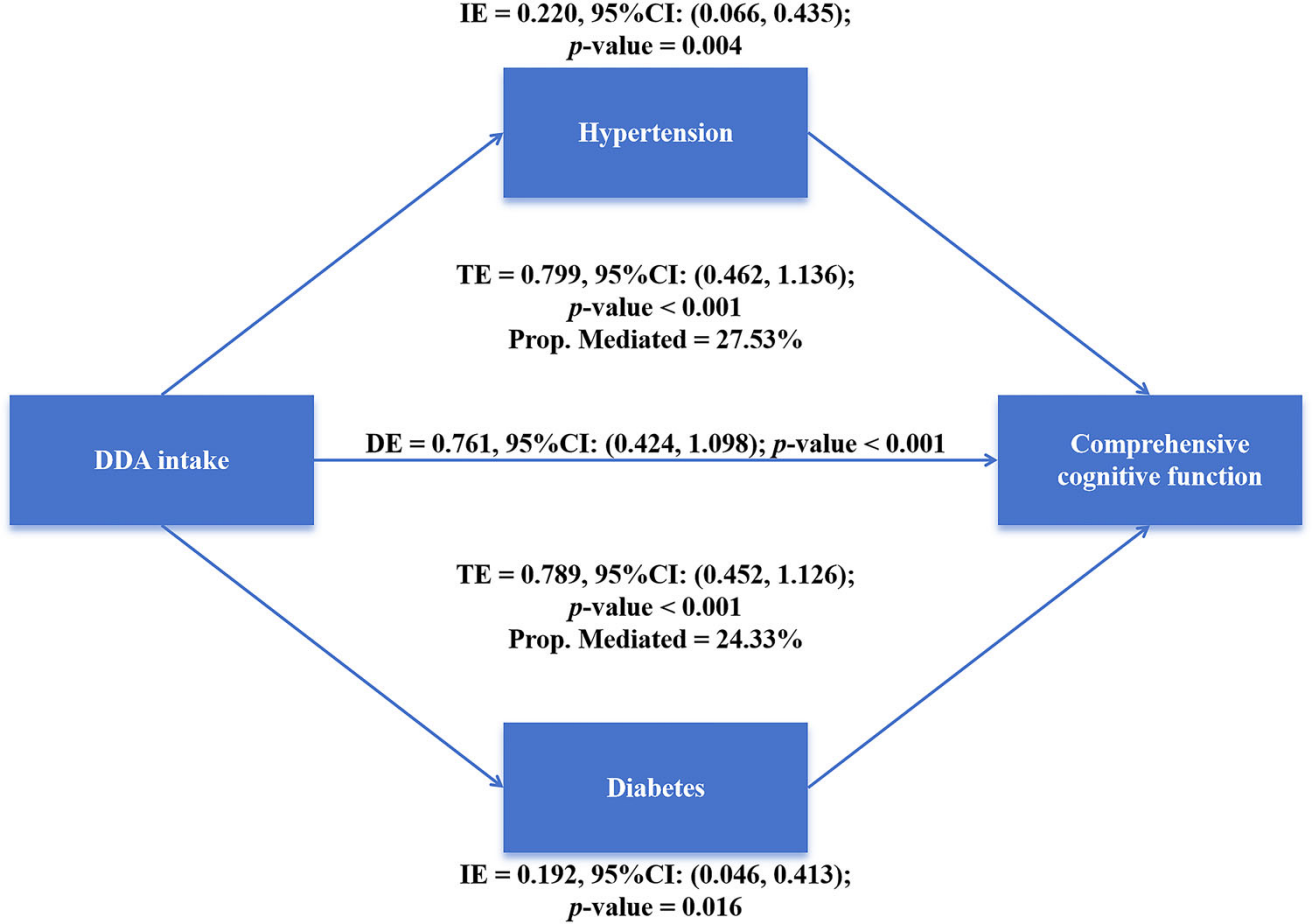
The mediating effects of hypertension and diabetes on the relationship between DDA intake and comprehensive cognitive function. Abbreviations: DDA, dietary decanoic acid; TE: total effect; DE: direct effect; IE: indirect effect. The mediating analysis adjusted for gender, age, race, education level, economy, marital status, BMI, WC, TC, TG, drinking status, smoking status, stroke, hypertension, and diabetes.

In the mediating analysis, we explored whether hypertension and diabetes were statistically associated as putative mediators (Table 3). The results showed that DDA intake was negatively correlated with hypertension (*β* = –0.470, *p* = 0.002) and diabetes (*β* = –0.463, *p* = 0.006), while hypertension and diabetes were negatively correlated with comprehensive cognitive function (hypertension: *β* = –0.510, *p* < 0.001; diabetes: *β* = –0.445, *p* = 0.001). The total effect of DDA intake on comprehensive cognitive function was 0.799 (*p* < 0.001) and 0.789 (*p* < 0.001), respectively; the direct effect was 0.761 (*p* < 0.001); and the indirect effect through hypertension and diabetes was 0.220 (*p* = 0.004) and 0.192 (*p* = 0.016), respectively. The indirect effects of hypertension and diabetes respectively accounted for 27.53% and 24.33% of the total effects (Figure 5).

**TABLE 3:** Mediating analysis of hypertension and diabetes in the association between DDA intake and comprehensive cognitive function.

Pathways	*β* (95% CI)	*p*‐value
Hypertension ∼ X	−0.470 (–0.773, –0.166)	0.002
Y ∼ Hypertension	−0.510 (–0.790, –0.230)	< 0.001
Total effect	0.799 (0.462, 1.136)	< 0.001
Direct effect	0.761 (0.424, 1.098)	< 0.001
Indirect effect	0.220 (0.066, 0.435)	0.004
Diabetes ∼ X	−0.463 (–0.795, –0.132)	0.006
Y ∼ Diabetes	−0.445 (–0.706, –0.184)	0.001
Total effect	0.789 (0.452, 1.126)	< 0.001
Direct effect	0.761 (0.424, 1.098)	< 0.001
Indirect effect	0.192 (0.046, 0.413)	0.016

*Note*: The mediating analysis adjusted for gender, age, race, education level, economy, marital status, BMI, WC, TC, TG, drinking status, smoking status, stroke, hypertension, and diabetes. X represents DDA intake and Y represents comprehensive cognitive function.

### 3.6 Sensitivity Analysis

When only the intake on the first day was used for repeated analysis, the effect of the correlation between DDA intake and the comprehensive cognitive function of the elderly remained largely consistent (Table S1).

## 4 Discussions

This is the first population‐based NHANES study linking DDA intake to cognitive health in older adults. Due to the cross‐sectional design, this study was unable to make causal inferences. The findings suggest a positive correlation, where increased DDA intake is associated with enhanced comprehensive cognitive performance in the elderly. This association persists even after accounting for a range of potential confounding variables. The RCS curve analysis indicates a significant positive correlation between DDA intake and comprehensive cognitive function, while the gentle or downward trend at the end cannot be explained as the diminishing effect of DDA. The upper‐tail estimates should be interpreted with caution, as the wide confidence bands limit conclusions about a true biological plateau. Subgroup analysis showed that the correlation between DDA and comprehensive cognitive function was relatively consistent among different subgroups. The subgroup trends, although visually different, should not be overinterpreted in the absence of multiplicative interaction. Furthermore, hypertension and diabetes were identified as the significant mediators in the association between DDA intake and comprehensive cognitive function.

Previous research has provided evidence for a link between decanoic acid consumption and cognitive performance. A double‐blind, randomized controlled trial (RCT) conducted in China reported that Alzheimer's disease (AD) patients who received dietary supplementation with decanoic acid had markedly lower scores on the AD Assessment Scale‐Cognitive Subscale than those in the placebo group (*p* < 0.01) (Xu et al. 2020). In alignment with these findings, a double‐blind, prospective RCT in Japan observed significant enhancements in the immediate and delayed logical memory test outcomes among patients with cognitive impairment after a 12‐week intervention with a decanoic acid‐enriched formula (Ota et al. 2019). In a longitudinal study of Canadian subjects with cognitive impairment, their several cognitive outcomes, included the ability of memory, language, performance and processing speed, improved from baseline after 6 months of daily receiving decanoic acid‐containing emulsion beverage (Fortier et al. 2019). These findings may indicate that long‐term intake of decanoic acid may improve cognitive function. However, all these studies leave crucial gaps. Firstly, all these studies were conducted on the basis of human intervention or the additional use of supplements containing decanoic acid, which was divorced from the daily real dietary environment. Secondly, the sample sizes of these studies are very small. Finally, there is no clear path through which cognitive benefits are mediated. In contrast, our research analyzed 2246 elderly people, representing over 42 million elderly Americans. DDA is quantified through two 24‐h dietary recalls, capturing the real‐world intake distribution rather than a fixed supplement. This mediation model further indicates that the association between DDA and cognitive function may be achieved by reducing hypertension and diabetes, which has not yet been verified in previous RCTs.

Currently, the physiological mechanism of the association between high DDA intake and good cognitive function has not been fully clarified. The cognitive decline may be related to the accumulation of beta‐amyloid (Aβ), excessive phosphorylation of tau protein (pTau), and synaptic dysfunction in the brain (Yang and Qiu 2024). The positive correlation between DDA and the cognitive function of the elderly may be associated with the remission of these lesions. As one of MCFAs, the hydrophilicity and short carbon chain of decanoic acid allow for direct entry into the liver and mitochondria without the need for carriers and carnitine systems (Jadhav and Annapure 2023). Decanoic acid undergoes rapid β‐oxidation in mitochondria to generate ketone bodies (KBs). Compared with glucose, KBs has higher energy when utilized (Pan et al. 2002). Under long‐term fasting, KBs levels significantly increase and become the main source of energy for the brain. Under non‐fasting conditions, increasing intake of MCFAs can also rise KBs levels (Puchalska and Crawford 2017). An animal experiment found that a long‐term ketoester diet have been associated with reduced Aβ deposition and pTau levels in specific regions of the mouse brain (Kashiwaya et al. 2013). KBs, especially β‐hydroxybutyrate (BHB), may act as antioxidants and may alleviate neuroinflammation and neurodegeneration by eliminating free radicals in the body and reducing oxidative damage (Nazzi et al. 2024; Wang et al. 2021). Inflammation is an important factor in cognitive decline and KBs can reduce neuroinflammation by suppressing the nuclear kappa‐B (NF‐κB) signaling pathway to reduce the synthesis of pro‐inflammatory cytokines, including interleukin‐1 beta (IL‐1β), interleukin‐6 (IL‐6), and tumor necrosis factor‐alpha (TNF‐α) (Taggart et al. 2005; Koh et al. 2020). Studies have shown that KBs have been associated with neurogenesis in the hippocampus, a key structure in the brain closely related to cognitive function (Saito et al. 2022; Mattson and Arumugam 2018). Experiments on rats have found that BHB has been linked to protection of hippocampal neurons from the toxicity of Aβ (Kashiwaya et al. 2000). In addition, decanoic acid crosses the blood‐brain barrier, a property hypothesized to be linked to cognitive correlates (Wlaź et al. 2012; Castellano et al. 2015).

Synaptic dysfunction is one of the early features of cognitive decline, in which α‐amino‐3‐hydroxy‐5‐methyl‐4‐isoxazolepropionic acid (AMPA)‐type glutamate receptor (AMPAR) plays an important role (Taoufik et al. 2018). AMPAR is essentially a Ca^2+^ permeable channel that regulates most of the fast excitatory synaptic transmission (Greig et al. 2000). The overactivation of AMPAR has excitotoxicity, leading to dysregulation of synaptic formation and neuronal damage (Joshi et al. 2015). Decanoic acid has been found to bind non‐competitively to AMPAR (Chang et al. 2016). In an animal experiment using an in vitro rat hippocampal slice model, it was found that decanoic acid binds to a site on the M3 helix of the AMPA‐GluA2 transmembrane domain, directly inhibiting excitatory neurotransmission in hippocampal slices (Chang et al. 2016). In the AD mice model, decanoic acid was able to rescue the AMPA mediated calcium elevation differences in astrocytes and neurons in the hippocampal CA1 region, indicating that decanoic acid has been linked to better cognitive performance correlating with altered glutamate‐mediated signaling (Abghari et al. 2023).

The results of the mediating analysis suggest that hypertension/diabetes may partially mediate the association between DDA intake and the comprehensive cognitive function of the elderly. Hypertension is a common cardiovascular disease and is increasingly recognized as a harmful factor to cognitive health (Tadic et al. 2016). A cross‐sectional study found a significant correlation between the history, duration, and severity of hypertension in elderly individuals and cognitive impairment (Anto et al. 2019). Hypertension leading to decreased cognitive function is the result of an imbalance in cerebral blood flow autoregulation and cerebrovascular disease (Tadic et al. 2016). Long‐term elevated blood pressure may lead to reduced blood flow in certain regions regulating learning and memory function via narrowing the cerebral vascular lumen and reducing elasticity (Alosco et al. 2014). Hypertension may lead to white matter lesions, which are associated with cognitive decline, particularly in executive function and processing speed (Petrea et al. 2024). Research indicated that reduced white matter is correlated with heightened risks of cognitive decline and dementia (Raz et al. 2012). Elevated blood pressure could contribute to a proliferation of neurofibrillary tangles within the brain, a hallmark of the neuropathology associated with cognitive impairment (van der Flier et al. 2018; Wharton et al. 2019). The formation of these tangles is the result of the accumulation of abnormally phosphorylated tau proteins within neurons (Drummond et al. 2020). Decanoic acid has been associated with lower hypertension, since KBs may improve endothelial cell function. KBs have been linked to reduced SBP and protect renal function in hypertensive rats (Chakraborty et al. 2018). Long‐term ketogenic diet can regulate gut microbiota to increases the expression of endothelial nitric oxide synthase (eNOS) in cerebral vascular, enhancing neurovascular function (Ma et al. 2018). Diabetes is considered to be one of the important factors affecting the cognitive function of the elderly (Zilliox et al. 2016). Research has shown that higher blood sugar levels are associated with poorer cognitive performance (Casagrande et al. 2021). Chronic hyperglycemia may induce activation of microglia and release inflammatory cytokines and promote neuroinflammation, leading to central nervous system damage and metabolic abnormalities, thereby deteriorating cognitive function (Nagayach et al. 2022). Insulin is also crucial for neuronal survival and brain function, and its receptors are widely distributed in cognitive related areas of the brain. Insulin resistance can lead to neuronal dysfunction and cognitive decline (Chen et al. 2022). Diabetes is associated with a variety of vascular complications, which may indirectly affect the blood supply and metabolism of the brain, thereby affecting cognitive function (Low et al. 2020). Decanoic acid may promote the secretion of glucagon like peptide‐1 (GLP‐1) by activating the receptor GPR84, a pathway linked to enhanced glucose tolerance and improved insulin resistance (Nonaka et al. 2022). In addition, decanoic acid exerts anti‐inflammatory properties by curbing the phosphorylation of mitogen‐activated protein kinase (MAPK) and the activation of NF‐κB, thereby reducing the secretion of IL‐6, IL‐8, and TNF‐α (Huang et al. 2014).

This study has some obvious advantages. Firstly, this study used data from NHANES 2011–2014 covering 2 cycles for a total of 4 years. This is a nationally representative large‐scale sample database that ensures the accuracy of research results. Secondly, we established multiple models and adjusted for various potential confounding factors in order to reduce interference from other factors. However, there are also some limitations that cannot be ignored. In this study, DDA intake was evaluated through two 24‐h dietary reviews. Although this method is widely used in large‐scale health surveys, it relies on participants’ memory and reporting, which may introduce recall and reporting biases. Two 24‐h recollections can only capture limited daily changes, especially for nutrients consumed irregularly. The DDA intake is occasional and not a regular nutrient, so daily differences dominate the observed inter‐individual variations. Participants may underestimate or overestimate the intake of certain foods, especially those they consider unimportant or infrequently consumed. For instance, some participants might forget to report the intake of small amounts of high‐DDA foods (such as coconut oil or dairy products), thereby leading to an underestimation of DDA intake. Based on only two reviews, non‐differential misclassification is inevitable, which will dilute the true relevance to invalid values. Therefore, due to recall bias, exaggerated or conservative results may be obtained. Given that our study is based on a single cross‐sectional survey, exposure, mediators and outcomes are measured simultaneously. It is very difficult to determine their sequence and to rule out the influence of unmeasured confounding factors. The so‐called “mediating proportion” can only quantify the statistical overlap between variables, rather than the mediating weights in the causal chain. Therefore, both the total effect and the indirect effect reported in our research should be regarded as “hypothesis generation” rather than definite causal relationships. In the future, longitudinal studies or intervention studies should be conducted to further verify and explore. In addition, despite extensive covariate adjustments, there may still be unmeasured confounding factors, such as dietary quality, overall fatty acid intake, and physical activity. If these unmeasured lifestyle factors are associated with DDA intake and cognition, they may amplify the observed association. Finally, NHANES provided representative data from the United States, but the main dietary sources of decanoic acid vary globally. Therefore, caution should be exercised when extrapolating research conclusions to populations in other countries.

## 5 Conclusions

Our study identified a positive association between increased DDA intake and improved cognitive performance. Both hypertension and diabetes may partially mediate this relationship. Cross‐sectional studies are difficult to determine the sequence and causal relationship. In the future, prospective studies or intervention studies should be conducted to explore further effects.

## Author Contributions

HZ: Designed the research plan, collected and analyzed data, explained the research results, and wrote the initial and final drafts. QF: Participated in research design, data analysis, and manuscript revision. JH: Participated in research design and manuscript revision. YW: Participated in data collection and analysis. JM: Supervised the research project, provided financial support, guided the research direction and content, and reviewed the final manuscript. All authors have given approval to the final version of the paper.

## Funding

This research was funded by Technology Project of Jiangxi Provincial Administration of Traditional Chinese Medicine (NO. 2023B1277).

## Conflicts of Interest

The authors declare that the research was conducted in the absence of any commercial or financial relationships that could be construed as a potential conflict of interest.

## Ethics Statement

This study utilized data from NHANES project that were publicly available and approved by National Center for Health Statistics (NCHS) Ethics Review Board (ERB). All participants provided informed consent before enrollment.

## Supporting information




**Table S1**: brb371180‐sup‐0001‐tableS1.docx

## Data Availability

The datasets analyzed during the current study are available in NHANES project (https://wwwn.cdc.gov/nchs/nhanes/Default.aspx).
