# Supplementary material for: Associations of Dietary Decanoic Acid Intake With Cognitive Function in the Elderly and the Mediating Effects of Hypertension and Diabetes: An Analysis From NHANES 2011–2014
Source: Brain Behav. 2026 Jan 28;16(2):e71180. doi: 10.1002/brb3.71180 (PMC12848519; doi:10.1002/brb3.71180)
Supplement: Supplementary file 1 — Table S1: brb371180‐sup‐0001‐tableS1.docx [file BRB3-16-e71180-s001.docx]

Table S1. Multiple linear regression analysis of DDA intake on the first day and cognitive function.

| DDA | Model 1 | | Model 2 | | Model 3 | |
| --- | --- | --- | --- | --- | --- | --- |
|  | β (95% CI) | *p*-value | β (95% CI) | *p*-value | β (95% CI) | *p*-value |
| **IRT Z-scores** | | | | | | |
| Continuous | 0.145(0.056, 0.235) | 0.002 | 0.120(0.027, 0.213) | 0.014 | 0.101(0.003, 0.200) | 0.044 |
| Q1 | Ref |  | Ref |  | Ref |  |
| Q2 | -0.087(-0.222, 0.049) | 0.2 | -0.124(-0.250, 0.002) | 0.053 | -0.125(-0.252, 0.003) | 0.054 |
| Q3 | 0.082(-0.055, 0.219) | 0.23 | 0.052(-0.073, 0.176) | 0.398 | 0.041(-0.087, 0.168) | 0.504 |
| Q4 | 0.117(-0.017, 0.250) | 0.084 | 0.068(-0.075, 0.211) | 0.338 | 0.050(-0.095, 0.196) | 0.471 |
| P for trend |  | 0.016 |  | 0.074 |  | 0.125 |
| **DRT Z-scores** | | | | | | |
| Continuous | 0.117(0.005, 0.229) | 0.041 | 0.102(-0.015, 0.219) | 0.085 | 0.086(-0.030, 0.202) | 0.135 |
| Q1 | Ref |  | Ref |  | Ref |  |
| Q2 | -0.114(-0.244, 0.015) | 0.082 | -0.143(-0.272, -0.015) | 0.03 | -0.143(-0.264, -0.021) | 0.025 |
| Q3 | 0.056(-0.080, 0.192) | 0.409 | 0.042(-0.085, 0.169) | 0.497 | 0.028(-0.093, 0.150) | 0.628 |
| Q4 | 0.068(-0.078, 0.213) | 0.349 | 0.036(-0.113, 0.185) | 0.621 | 0.019(-0.126, 0.163) | 0.784 |
| P for trend |  | 0.119 |  | 0.2 |  | 0.284 |
| **AFT Z-scores** | | | | | | |
| Continuous | 0.232(0.068, 0.396) | 0.007 | 0.088(-0.077, 0.254) | 0.282 | 0.067(-0.099, 0.234) | 0.403 |
| Q1 | Ref |  | Ref |  | Ref |  |
| Q2 | 0.109(-0.072, 0.290) | 0.228 | 0.007(-0.143, 0.158) | 0.919 | 0.004(-0.160, 0.169) | 0.956 |
| Q3 | 0.182(-0.000, 0.364) | 0.05 | 0.061(-0.113, 0.234) | 0.478 | 0.051(-0.128, 0.230) | 0.551 |
| Q4 | 0.312(0.081, 0.543) | 0.01 | 0.120(-0.107, 0.346) | 0.286 | 0.100(-0.140, 0.340) | 0.386 |
| P for trend |  | 0.006 |  | 0.224 |  | 0.312 |
| **DSST Z-scores** | | | | | | |
| Continuous | 0.196(0.087, 0.305) | <0.001 | 0.095(-0.004, 0.193) | 0.058 | 0.069(-0.034, 0.171) | 0.175 |
| Q1 | Ref |  | Ref |  | Ref |  |
| Q2 | 0.085(-0.038, 0.209) | 0.169 | -0.007(-0.098, 0.084) | 0.88 | -0.011(-0.108, 0.086) | 0.815 |
| Q3 | 0.117(-0.042, 0.276) | 0.143 | 0.015(-0.115, 0.145) | 0.817 | 0.008(-0.107, 0.123) | 0.882 |
| Q4 | 0.266(0.110, 0.421) | 0.002 | 0.114(-0.015, 0.243) | 0.08 | 0.088(-0.046, 0.223) | 0.181 |
| P for trend |  | 0.002 |  | 0.075 |  | 0.154 |
| **Comprehensive Z-scores** | | | | | | |
| Continuous | 0.690(0.379, 1.001) | <0.001 | 0.405(0.079, 0.732) | 0.017 | 0.324(-0.007, 0.655) | 0.055 |
| Q1 | Ref |  | Ref |  | Ref |  |
| Q2 | -0.007(-0.460, 0.446) | 0.976 | -0.267(-0.619, 0.085) | 0.13 | -0.274(-0.635, 0.088) | 0.127 |
| Q3 | 0.437(-0.045, 0.920) | 0.074 | 0.169(-0.257, 0.596) | 0.42 | 0.128(-0.276, 0.532) | 0.508 |
| Q4 | 0.762(0.259, 1.264) | 0.004 | 0.337(-0.157, 0.831) | 0.172 | 0.258(-0.254, 0.769) | 0.298 |
| P for trend |  | 0.001 |  | 0.056 |  | 0.112 |

Model 1 is unadjusted. Model 2 adjusts for gender, age, race, education level, economy, and marital status. Model 3 adjusts for gender, age, race, education level, economy, marital status, BMI, WC, TC, TG, drinking status, smoking status, stroke, hypertension and diabetes.
